# Consumption of Fruits and Vegetables by Low-Income Brazilian Undergraduate Students: A Cross-Sectional Study

**DOI:** 10.3390/nu10081121

**Published:** 2018-08-19

**Authors:** Ygraine Hartmann, Raquel B. A. Botelho, Rita de Cássia C. de A. Akutsu, Renata Puppin Zandonadi

**Affiliations:** Department of Nutrition, University of Brasília, Brasília 70910-900, Brazil; ygrainehartmann@gmail.com (Y.H.); raquelbabotelho@gmail.com (R.B.A.B.); rita.akutsu@gmail.com (R.d.C.C.d.A.A.)

**Keywords:** consumption of fruits and vegetables, low-income undergraduate students, Student Assistance Program

## Abstract

**Objective:** This study aimed to evaluate the consumption of fruits and vegetables (FV) by low-income students participating in the Brazilian Student Assistance Program. **Methods:** For three days, we measured participants’ consumption through direct observation of food intake at the University Restaurant (UR) and 24-h recall outside the restaurant. The 174 undergraduates were divided into two groups to obtain data on FV intake at the weekend (Sunday) and two days of the week. Group 1 included low-income undergraduates who received their meals for free, and Group 2 included students who paid for their meals at the UR. **Results:** Both groups presented a very low consumption of FV. On the weekend, Group 1 consumption was equal to Group 2, but it was higher than Group 2 on weekdays, demonstrating how important the UR is for this population. The lowest contribution of the UR to the daily consumption of FV was 59%, reaching a percentage of 87.27%. Fruit supply in the restaurant menu may have positively influenced this consumption. **Conclusions:** The consumption of FV varied according to the menu offered at the UR. The UR should be a space to promote healthy eating habits including more FV in its menus.

## 1. Introduction

Fruit and vegetable (FV) consumption is associated with several health advantages, such as lowered chronic diseases because of their low energy density, high fiber, bioactive compounds, vitamins, and minerals profile [1]. Studies [2,3,4,5] have shown that the population’s consumption of FV does not meet the recommendations of the World Health Organization (WHO) (400 g per day) [6]. In some countries, socioeconomic disparities of diet quality are well established. Low-income and low levels of education have been associated with poor diets and poor health [7].

Multiple factors are known to influence diet quality, including economic barriers, inadequate nutrition knowledge and awareness, food preferences and attitudes, and cultural factors [7]. The decision to consume FV, mainly out of the home, can be influenced by several factors, including confidence in the hygiene of served raw products (salad), variety, attractiveness, sensory balance, and appearance [8]. Some studies have shown the association of physical distance to a supermarket or restaurant with FV intake or overall diet quality and body weight [7,9,10,11]. Therefore, ensuring physical access to FV suppliers has recently become the focus of public health policies designed to improve the diets and health [7,9,10,11,12] of low-income population.

Cook and Papadak [13], studying United Kingdom university students, showed that knowledge influences the FV consumption. Students with higher knowledge level tended to get involved in healthier eating practices, including higher consumption of FV [13]. Divergently, a study conducted in Tunisia with university students showed an inadequate consumption of FV, and 37% of the students were overweight, and 9% were obese [14]. The eating habits of young people can inform us about the future demand for food, as well as the possibility of certain diseases increasing. This knowledge is relevant to design nutritional policies on health promotion [15].

In Brazil, the Brazilian Family Budget Survey (POF) revealed that less than 10% of the population meets the recommendations of FV intake [16]. As part of public health policies designed to improve low-income diets and health, there is a National Program of University Student Assistance (PNAES), in which the low-income students have free access to food at the University Restaurants (URs) [12] to guarantee the social rights of feeding, consolidated by the Universal Declaration of Human Rights [17]. To participate in this Program, students of the Brazilian public universities should be primarily from public schools. Also, their average family income should be of up to 1.5 minimum wage. At the University of Brasilia, this program offers free breakfast, lunch, and dinner, from Monday to Saturday. The UR’s meals should be nutritionally balanced, with a basic composition of fruits, raw vegetables, white and brown rice, baked bean, cooked vegetables, and protein dishes. Meals at the UR are free for students of the PNAES, while for other students, it has a cost of US$0.83 per meal [18].

It is essential that a UR subsidized by the government offers different options of FV to its students, to improve their FV consumption and to achieve the WHO’s target [6]. Menu planning is the key point for this offer to be conducted in ways that encourage FV consumption. Nutrition education strategies are also important, for students to be not only exposed to FV, but to consume them. Therefore, this study aimed primarily to evaluate the consumption of FV by students participating in the Student Assistance Program who regularly eat at the UR of the University of Brasilia. We also aimed to compare them to a group of students who are not part of the program, as well as to assess the UR’s contribution to the daily intake of FV.

## 2. Materials and Methods

### 2.1. Study Design

We conducted this study at the UR of the University of Brasilia, Brazil. We chose a matched cross-sectional epidemiological method with the allocation of two groups through the control of the variables age (±2 years) and sex, to nullify the confounding variables. This increased the possibility of participants to present similar characteristics, as well as the power of the statistical tests to detect differences or associations with the objective to make easier interpretation of the results.

We gathered data for three months to complete the entire protocol. We observed each participant for two consecutive days (weekdays) during meals at the UR and on Sunday, we used 24-h recall for the whole day consumption assessment. The Health School Ethics Committee of the University of Brasilia approved this study (Report No. 610.774/2014).

### 2.2. Subjects

Group 1 consisted of low-income students who use the UR free of charge—support provided by the university to guarantee low-income student’s food access, and permanence and graduation at the university. We used the UR’s access control system to obtain data to set this group. Group 1 presented an average family income of up to 1.5 minimum wage (about US$435.00), one of the criteria created by the university for students to receive assistance. Criteria for inclusion of individuals in this study were to have free access to food at the UR, as part in PNAES assistance program; to have had two meals on the same day at the UR, at least once, within fifteen days prior the start of the study; the student’s consent to take part in the research; to be at least 18 years old.

Group 2 included students from the university who do not participate in PNAES, but they go to the UR to have meals, either lunch or dinner, independently of the frequency. Group 2 had a family income higher than 1.5 minimum wage since they are not part of PNAES. We excluded pregnant women from this study, since they had different nutritional needs and body composition from the general population. Students from Group 2 also needed to be at least 18 years old and to agree to take part in the research.

In Group 1 (low-income assistance students), 439 individuals received our email, inviting students to be a participant and to answer a short questionnaire that contributed to the design of their socioeconomic profile. We used the SurveyMonkey^®^ tool to gather information. All the individuals that answered the questionnaire were invited to be part of the follow-up research phase at the UR. The final sample for this group was 79 individuals that completed the two-days meals’ observation and one-day 24-h recall.

In Group 2 (non-assistance students), students were randomly selected from the cafeteria line, at the entrance of the UR, who accepted to participate in the study. The size of the sample was the same as Group 1 (*n* = 439). However, during the research, students who accepted participating while in line on the first day, did not come to the UR to complete the three days established in our protocol. Therefore, the final sample for this group was 94 individuals. The selection criteria at the cafeteria line followed the methodology used by Godoy et al. [19], using a systematic order to approach users, one user for every 15 that entered the UR on the day of data gathering. When the researchers achieved the final sample, they did not invite more students to participate.

Group 2 answered the same questionnaire as Group 1 after acceptance to participate in the research on the first day of observation. Students from this group received information that they needed to come to the UR for a second and third day, and they should identify themselves to researchers at the area designed for data gathering.

Students from both groups who agreed to participate had their meals in an exclusive dining room. Their meals could consist of breakfast, lunch, and/or dinner, that were offered at the UR from Monday to Saturday. The UR represents six cafeterias divided into three different floors. To enter the UR, students enter a unique line, and when inside the main floor, they may choose their way to one of the cafeterias. Therefore, researchers approached students at the main floor to invite them for the study. Afterward, researchers conducted students at one of the cafeterias in other to gather all the participants in the same room. This procedure was important to observe the plate’s assembly.

### 2.3. Variables and Instruments

The socioeconomic variables chosen for this study were: sex, age group, and income. Besides these data, we measured the weight and height for Group 1 and Group 2. For anthropometric evaluation, we collected measurements of weight (G-tech scale, model: 200 Glass of 200 Kg capacity) and height and, calculated the body mass index (BMI). We classified the BMI following the criteria adopted by the World Health Organization (WHO) [20]. Students who agreed to participate were invited to take part in this part of the study. Researchers provided different times and dates for students to come to the UR to take the measurements. To encourage this procedure, we informed the students that nutritionists would be available on the same day to give orientations.

To verify the consumption of each group, we applied 24-h recall for the weekend day and meals consumed outside the UR and direct observation for meals performed at the UR. Through the 24-h recall, it was possible to calculate the average number of meals performed by each group.

The assembly of meals was at the students’ discretion, who were free to choose the amount of food that suited them. For breakfast, all the items on the menu were portioned, and students could be served, or not, one portion of each item. For lunch and dinner, students served themselves for the following items: rice, beans, cooked vegetables, raw salads, soup, bread, spices, olive oil, sauces, and cassava flour. For main course (protein dish) and fruit or sweet desserts, an employee of the UR portioned them every day for all the students. It is important to highlight that the students served vegetables (raw or cooked), and they could decide their portions for lunch and dinner. The employee of the UR portioned the fruit, and served students who wanted to get the fruit. Students were free to get the fruit served, as well as being able to reject FV. They could ask the employee to have a smaller serving of the fruit too, but they could not ask for more. This serving procedure is a limiting factor for fruit consumption, but the comparison is still important because students could refuse to get the fruit, they could ask for smaller portions, and they could consume fruits in other meals outside the UR. We compared the fruit consumption considering the whole day consumption for both groups.

This practice already occurred at the University Restaurant, and the researchers preserved it in this research because of the cost of these ingredients, and the amount of protein calculated for a balanced meal. Trained observers accompanied meals’ assembly by previously classifying the portion sizes served by the students according to the methodology proposed by Savio et al. [21]. This methodology consists of prior weighing of portion sizes before the UR is open to the public. With the utensils presented to the students at the cafeteria, observers weighed three times small, medium, large, and extra-large portions. We invited students to participate, but they did not know that we were analyzing the intake of FV. Observers did not interfere in meal assembly. As a student walked through the food line, the observer classified each portion of each component of the meal.

We weighed the served meal (Filizola^®^, 20 kg capacity, Brazil) and the weight was recorded on the appropriate manner, containing the student’s identification, the weight of the empty plate, the weight of the plate with the food, and the weight of the plate with leftovers. Other weighed items were those served out of the plate composition, such as salads and soups served separately, drinks, desserts, bread, spices, olive oil, sauces, and cassava flour. When the individual returned for a second serving, we repeated the observation and weighing procedure, and we considered them for the assessment of one’s consumption. Both groups could have a second serving, except for the protein dish and fruits or desserts for lunch and dinner. For breakfast, the UR did not allow a second serving.

For meal leftovers, we discounted them proportionally by the items placed on the plate for every meal held at the UR. Therefore, plates had to be returned to a specific area in the cafeteria, and the researchers weighed them.

While eating their meals at the UR, the researchers asked the students about the food consumed outside the UR by using a 24-h recall (R24h) during the two weekdays of data collection. To evaluate food consumed on one day of the weekend (Sunday), we used the R24h to gather the individual’s food consumption out of their routine. Therefore, we obtained three complete days of consumption for both groups. 

To evaluate the intake of FV, we considered these foods separately; that is, those identified by direct observation at the UR and those that were described by the students with their respective amounts reported in the R24h. This separate data was important to evaluate FV intake outside and inside the UR. We excluded the consumption of starchy vegetables (potatoes, cassava) from the daily intake of vegetables. It is a methodology used by the WHO [6], that also excludes these foods from their recommendation of 400 g of FV per day. The researchers recorded all FV consumed during the day, excluding juices, smoothies (fruits blended with milk), and stewed meat with vegetables, due to the inaccuracy of measurement. In both groups, only four students consumed smoothies (not recorded data).

In the end, for each student, we had the following data to analyze and compare groups: body weight, height, body mass index, answered questionnaire, one-weekend day 24-h recall intake, two complete weekdays’ intake using 24-h recall for meals outside the UR, and direct observation of meals consumed at the UR considering leftovers.

### 2.4. Statistical Treatment

We entered and processed data into a database specifically developed for this study using the Statistical Package for Science Program—SPSS version 20.0^®^. After the creation of the data input form, we checked them through frequency distribution analysis, comparing the values of each variable in the SPSS database to those of possible occurrence, preventing typos. Normality assumptions were checked (Kolmogorov–Smirnov), and we determined the measures of central tendency and sample variance. To verify the differences between the studied groups, we used the *t* test for means, Kruskal–Wallis and the Mann–Whitney tests along with Tukey’s post hoc procedure for proportions. To evaluate the correlation among variables and the difference in consumption between groups by sex, we applied Pearson’s chi-square test.

## 3. Results

The average number of meals eaten by both groups is 3.58 ± 0.96. The most common meals were breakfast, lunch, and dinner. When evaluating each group separately, Group 1 had an average number of meals of 3.51 ± 0.93, and Group 2 of 3.64 ± 0.99. Analyzing by group and sex, women from Group 2 presented the highest number of meals (3.94 ± 0.95). The lowest number of eaten meals was 3.40 ± 0.97 for men in Group 1.

Table 1 shows the *n* and the percentage of the anthropometric profile, socioeconomic, and demographic variables of the final samples in each group. Table 2 shows FV intake by sex and group of students by quartiles due to the large variance of intake, since we did not confirm normality assumptions. The consumption of FV on Sunday did not reach the 400 g recommendation in any of the groups (Table 2).

We had similar groups for comparison of data. All students from Group 1 had low family income as stated in the inclusion criteria. For Group 2, most of the students had an income above five minimum wages, showing better conditions to improve their diets.

In Group 1, we did not identify a correlation between BMI and the consumption of FV (*p* = 0.784). However, in Group 2, among women, for Day 2, consumption showed a negative correlation between the consumption of FV and BMI, with *p* = 0.04 and a correlation of 0.452. According to average BMI, groups mainly presented the normal classification, but means were statistically different. Group 1 presented more low weight students and no individuals classified as obesity 2.

The intake of FV on Day 2 was higher than on the weekend, but none of the groups achieved the recommendation level. On Day 3, the students of Group 1 in the third quartile, concerning both genders, exceeded the recommended level. The prevalence of consumption adequacy (≥400 g/day) is in Table 2.

Table 3 presents the prevalence of FV consumption by the group for each day of analysis. Consumption of Group 1 on Day 3 was higher than Group 2 with statistical difference (*p* = 0.001; Mann–Whitney *U* test), for Days 1 and 2, there was no statistical difference (*p* = 0.105—day1; *p* = 0.465—day2). When comparing both groups for the three days of the study, Group 1 consumed higher quantities of FV than Group 2 for all the quartiles (*p* = 0.001). It is important to highlight that percentiles demonstrate the data since we did not confirm normality assumptions.

There was a significant difference (chi-square) concerning the consumption proportion of FV between the groups by sex only for Group 2 on Day 2 (*p* = 0.002) (Table 4). Considering the average consumption of the two samples, on Day 3, only Group 1 achieved the recommendation of 400 g of FV. The average consumption of fruits (144.59 g/day) of the entire sample (*n* = 173) was higher than the consumption of vegetables (60.98 g/day).

The percentage contribution of the UR to the daily consumption of FV is in Table 5. It reveals that the contribution of the restaurant is significant, with 59.9% consumption by women of Group 1 on Day 2, and a higher contribution among men of Group 1 on Day 3, reaching 87.3%.

On Day 3, the UR’s menu offered more FV, as shown in Table 6. On this day, fruits were the dessert during lunch, unlike Day 2, when the UR offered a sweet dessert. A larger watermelon portion (350 g) for breakfast, and the offer of an apple (126 g) as a dessert for lunch, had a positive effect on student’s consumption, presenting statistical differences between groups on this day.

## 4. Discussion

Both groups are similar, which allows comparing data by sex. Groups presented lower percentage of overweight (group 1–10.1%, group 2–12.8%) and obese (group 1–5.1%, group 2–3.2%) when compared to the Brazilian population (50% of overweight; 12% of obesity) [22].

The average number of daily meals eaten by both groups is 3.58, which may be negatively influencing the daily consumption of fruits. Brazilians tend to consume fruits mostly in the form of snacks during breaks, and they are often encouraged by nutritionists to eat fruits at these hours as well.

In this study, the median of FV intake was higher in the days that the participants ate at the UR than the median of FV intake on weekends for both groups. However, Group 1 consumption of FV on weekends did not differ from Group 2, whereas Group 1 consumption of FV was higher than Group 2 in one of the days that the groups ate at the UR. The prevalence of FV intake reached 39% among men on the day that the UR’s menu offered fruits as dessert for lunch and dinner. Therefore, it is essential to highlight the importance of good menu planning and how the UR can positively contribute to the students’ food choices. Since fruit portions are limited to students, this intake could be even higher at the UR. However, vegetable intake was much lower, and students were free to serve themselves from this group of food.

Several studies undertaken in different countries [5,8,13,14,23,24] showed a low prevalence of regular consumption of FV by undergraduates. A recent study carried out at the University of Acre in the northern region of Brazil with 863 students showed a prevalence of regular consumption of FV in just 14% of the sample [24].

Among Turkish college students, the prevalence of inadequate intake of FV reaches 66.1% for men and 63.1% for women. The eating habits of Greek students also proved to be particularly poor regarding the consumption of FV and, consequently, 68.1% of men and 53.9% of women presented a fiber consumption rate below RDA recommendations [14].

In this study, regarding only the women of Group 2, the higher the BMI, the lower the consumption of FV, which did not favor weight loss in this group.

In another survey conducted in Brazil, only 24.9% of the subjects interviewed consumed adequate amounts of FV [16]. In another study, undergraduate students with regular consumption of FV presented a mean BMI = 23.2 (SD = 4.0) and revealed inadequate consumption patterns of these products [25].

In the present study, Day 3 showed the highest consumption of FV. The prevalence of adequacy of FV consumption was of 23% among men and 31% among women in Group 1, and in Group 2, the rate was 39% and 36% among men and women, respectively.

Therefore, the percentages found in this study are consistent with the results found in studies undertaken internationally. Food choices during this stage of life can be a determining factor regarding optimal health outcomes in the future and the appearance of Noncommunicable Chronic Diseases (NCD) [25].

Students of Group 1 had a higher contribution from the UR in the daily intake of FV than on the weekends, when meals occurred outside the UR. On Sunday, the consumption of FV was very low and did not reach the recommendation level. Also, on the weekend, Group 1 consumption was as low as Group 2, demonstrating how important the UR is for this population, since less healthy and more convenient choices occur on weekends. It is noteworthy that, in Brazil, the consumption of FV at home involves the steps of cleaning and sanitizing, which are tiring and may hinder the options of undergraduate students for these foods. Furthermore, they are more perishable, and must be consumed closer to the date of their acquisition. Besides, to consume FV on weekends, students from Group 1 must pay for them. In the UR, they receive FV without expenses. Therefore, the Student Assistance Program in Brazilian URs guarantees low-income students to have access to healthier meals than the ones eaten out of the university. Probably, it is related to the cost and food access for this group.

Since it is a low-income population, it is difficult for them to get important nutrients from FV in other forms, like nutraceuticals. Studies have shown the role of nutrients from plants in decreasing the incidence of cardiovascular problems, such as the research from Giordano et al. [26] studying carotenoids in FV, and from Metzger et al. [27] studying pectin, polyphenols, and phytosterols in lipid lowering. Scicchitano et al. [28] showed in their review that cardiovascular protection by nutraceuticals needs more debate. However, studies show that nutrients in FV present important benefits for people’s health, and they can be easily bought and consumed.

At the same time, we noted that by increasing exposure—i.e., the occurrence of FV in the UR menu—there is an increment on consumption, yielding a positive relation concerning intake.

In a study performed in Brazil, nutrition education actions on FV consumption that combined information and motivation were successful in impoverished environments [29]. The actions included providing knowledge about the advantages of consuming FV to achieve good health and enhancing the skills for its introduction in daily eating. It suggests that nutritional education programs would positively impact the students participating in the PNAES (Group 1).

It is noteworthy that the highest consumption of FV on Day 3 is due mainly to a higher offer of fruits and to the portion size of each of the fruits offered in the menu (banana—100 g; watermelon—350 g; apple—126 g, guava jelly—25 g).

A limiting factor in fruit consumption at the UR relates to the fact that the UR serves portioned fruits. This practice occurs because many users want to take a spare portion with them after leaving the UR. In other cases, not limiting fruit consumption can affect availability, which can lead to the lack of fruits for other students. One suggestion is that the UR improves the distribution of fruits during the week so that students receive greater portions and for all days of the week.

Another limiting factor is the number of researched days. More days could increase to verify the effect of the menu over FV consumption in both groups.

Considering that the consumption of fruits revealed to be higher than of vegetables, it is necessary to understand the reasons for the low consumption of vegetables and direct actions towards encouraging better food choices. Since it is a cross-sectional study, it has some limitations, which do not allow causal judgments among the associations.

Low confidence in food hygiene procedures may be one of the factors for the low consumption of raw vegetables offered in the menu [8]. Providing information about food cleaning procedures, implementing guided tours at the food area production, fostering the distribution of media newsletters, and implementing local campaigns at the cafeteria are actions that can increase student’s confidence, and thus, encourage consumption. People’s confidence is an important factor to improve good choices. Marinangeli and Jones [30] also showed that consumers would consume more nutraceuticals or functional foods if they can trust industrial procedures.

Nutrition education programs are essential for people to understand the importance of regular consumption of FV. It is necessary that the intake does not happen only at the UR, but during other meals and on weekends as well.

## 5. Conclusions

Based on the results, it is possible to affirm the great importance that the UR has over students’ food consumption. The fact that they spend most of their time at the university leaves them vulnerable to eating whatever is offered. The Student Assistance actions in Brazil for Group 1 and the low price of meals for Group 2 favor the presence of students and, therefore, requires that the UR becomes a place that fosters the promotion of health and life quality, adopting healthy eating habits that lead to the prevention of chronic diseases.

Students consume much lower amounts of FV than the ones recommended by the WHO, but on the day that the UR served larger portions of fruits for breakfast and desserts were fruits, Group 1 showed a closer median of intake to recommendations. When individuals consume FV, fruits are the largest portions, which demonstrate that vegetable consumption deserves greater incentive. Students could serve themselves vegetables as much as they wanted, but the offer is not the only criteria to encourage vegetable intake. Consumption was similar between male and female, and it was not related to BMI.

Proper menu planning with daily offers of FV is essential. At the same time, promotion of educational nutrition and healthy eating habits actions can help improving students’ food choices. It was possible to verify that, on the day, if the FV offer was more significant at the UR, the consumption was higher. The Student Assistance Program in Brazilian URs allows the low-income students to have access to healthier meals than the ones eaten out of the university. This result highlights the importance of the Food and Nutrition Policy developed by the universities through their URs.

## Figures and Tables

**Table 1:** Anthropometric profile, socioeconomic and demographic variables for both groups of students, consumers at the University Restaurants.

Variables		Group 1 (*n* = 79)		*p*	Group 2 (*n* = 94)		*p*
		*n*	%		*n*	%	
Sex	Male	45	57	0.216 ***	52	55.3	0.302 ***
	Female	34	43		42	44.7	
Age Group	<20	21	26.6		24	25.5	
	20–29	54	68.4	0.000 **	63	67	0.001 **
	30–39	3	3.8		5	5.3	
	>40	1	1.3		2	2.1	
BMI (average ± SD)	kg/m^2^	21.8 ± 3.7		0,027 ****	23.2 ± 4.2		0.027 ****
BMI (classification)	Low weight	14	17.7		3	3.2	
	Normal	52	65.8		35	37.2	
	Overweight	8	10.1		12	12.8	
	Obesity 1	4	5.1		1	1.1	
	Obesity 2	-	-		2	2.1	
						
Income	<1.5 mw *	79	100		-	-	
	>1.5–3 mw *	-	-	-	21	22.3	
	>3–5 mw *	-	-		10	10.6	0.001 **
	>5 mw *	-	-		28	29.8	
	Not Declared, but >1.5 mw	-	-		35	37.2	

* mw—Brazilian minimum wage (US$289.00); ** Kruskal–Wallis; *** Mann–Whitney test; **** *t* test.

**Table 2:** Fruit and vegetable intake by sex and group of students, consumers at the University Restaurant.

	Group 1 *n* = 89						*p* *	Group 2 *n* = 94						*p* *
	Male (*n* = 45)			Female (*n* = 34)				Male (*n* = 52)			Female (*n* = 42)			
	P25	Median	P75	P25	Median	P75		P25	Median	P75	P25	Median	P75	
	g	g	g	g	g	g		g	g	g	g	g	g	
FV intake weekend—Day 1	0	75	180	0	72.5	169.5	0.938	0	76	206	0	80	209	0.779
FV intake—Day 2	106	191	306	99.5	189.5	308	0.465	48	150	264	48	124	262	0.001
FV intake—Day 3	134	283	525	139.2	297	551.5	0.944	90	160	246.5	92.2	163.5	215.7	0.545

* Mann-Whitney U test.

**Table 3:** Fruit and vegetable intake by a group of students, consumers at the University Restaurant for each evaluated day.

	Group 1			Group 2			*p*
	P25	Median	P75	P25	Median	P75	
	g	g	g	g	g	g	
Weekend Day 1	0.0	100.0	196.0	0.0	76.0	180.0	0.465
Day 2	116.0	170.5	314.0	48.0	124.0	263.0	0.105
Day 3	207.0	370.0	626,00	44.0	173.0	353.0	0.000

**Table 4:** Percentage of students at the University Restaurant who consumed over four hundred grams of fruits and vegetables.

	Group 1 (*n* = 89)				*p* **	Group 2 (*n* = 94)				*p* **
	Male (*n* = 45)		Female (*n* = 34)			Male (*n* = 52)		Female (*n* = 42)		
	*n*	%	*n*	%		*n*	%	*n*	%	
Day 1 *	4	8.9	4	11.8	0.677	6	11.5	7	16.7	0.476
Day 2	5	11.1	4	11.8	0.928	11	21.2	0	0	0.002
Day 3	20	44.4	16	47.1	0.818	6	11.5	4	9.5	0.754

* Weekend day (Sunday), when the feeding did not occur at the University Restaurant (UR). ** Chi-square test.

**Table 5:** The contribution of the University Restaurant to the daily intake of fruits and vegetables by sex and group of students.

	Group 1			Group 2		
	Male (*n* = 45)	Female (*n* = 33)	Total	Male (*n* = 32)	Female (*n* = 42)	Total
Fruits and vegetable intake—Day 2	70.0%	59.9%	64.9%	69.4%	74.1%	71.7%
Fruits and vegetable intake—Day 3	87.3%	81.5%	84.4%	73.9%	67.4%	70.6%

**Table 6:** The qualitative offer of fruits and vegetables at the University Restaurant menu.

Day/Meal	Salad 1	Salad 2	Garnish	Dessert
Day 2 breakfast	-	-	-	Banana
Day 2 lunch	Crisp lettuce	Raw beet	-	Sweet guava pastes
Day 2 dinner	Cucumber	-	Braised cabbage	Pineapple
Day 3 breakfast	-	-	-	Watermelon
Day 3 lunch	Chard	Raw carrot	-	Apple
Day 3 dinner	Crisp lettuce	-	Braised chayote	Papaya
